# Pre-induction nociception indices are associated with post-induction hypotension during general anesthesia in older patients: a prospective observational study

**DOI:** 10.1007/s10877-026-01469-1

**Published:** 2026-07-14

**Authors:** Masahiro Kuroki, Rintaro Tsunoda, Tatsuya Hayasaka, Kenya Yarimizu, Ayuka Narisawa, Hiroaki Toyama

**Affiliations:** Department of Anesthesiology, Yamagata University Hospital, 2-2-2 Iida-nishi, Yamagata, 990-9585 Japan

**Keywords:** Analgesia Nociception Index, Anesthesia induction, Hypotension, Nociception Level, Nociception monitor

## Abstract

We investigated whether pre-induction High Frequency Variability Index (HFVI)/Analgesia Nociception Index (ANI) and Nociception Level (NOL) values could predict hypotension during general anesthesia induction in older patients. This prospective observational study included patients aged ≥ 65 years undergoing elective surgery under general anesthesia. After recording HFVI/ANI and NOL values, remifentanil was administered at 0.2 µg/kg/min for 2 min, followed by a rapid intravenous bolus administration of propofol at 2 mg/kg. Noninvasive blood pressure was measured at 1-min intervals over 5 min. Hypotension was defined as mean arterial pressure (MAP) < 65 mmHg. Predictive performance was evaluated using receiver operating characteristic (ROC) curves. Additionally, the area under MAP < 65 mmHg (AUT-65), a continuous metric integrating the depth and duration of hypotension, was calculated and correlated with nociception index values. We analyzed 51 patients (19 men; 22 in the hypotension group and 29 in the nonhypotension group). The hypotension group demonstrated lower HFVI/ANIi values (58.7 ± 11.1 vs. 69.0 ± 15.1, *p* = 0.010) and higher NOL values (17.2 ± 9.1 vs. 10.7 ± 8.8, *p* = 0.013). HFVI/ANIi (AUC = 0.72, cutoff = 62.5) and NOL (AUC = 0.71, cutoff = 13.5) moderately predicted hypotension. AUT-65 was significantly correlated with HFVI/ANIi (Spearman rho = − 0.35, *p* = 0.013) and NOL (rho = 0.47, *p* = 0.0005). Pre-induction HFVI/ANI and NOL were associated with the occurrence and cumulative burden of post-induction hypotension in patients aged ≥ 65 years, suggesting their potential utility as pre-induction screening tools.

*Trial registration* This study was approved by the Ethics Committee of Yamagata University Hospital on January 18, 2024 (approval number 2023 − 280). Before commencement, the study was registered as a prospective observational study in the UMIN Clinical Trials Registry (UMIN000053529).

## Introduction

Propofol and remifentanil, both widely used for anesthetic induction, possess vasodilatory properties and reduce peripheral vascular resistance, potentially causing hypotension. In clinical practice, propofol and remifentanil are frequently administered during induction of general anesthesia with endotracheal intubation to prevent adverse sympathetic reflexes and patient movement, which require deep unconsciousness, analgesia, and muscle relaxation. However, relatively excessive doses of sedatives or analgesics may lead to adverse events such as severe hypotension, particularly in older patients [1, 2]. Intraoperative hypotension is associated with severe organ dysfunction, including acute ischemic stroke and acute kidney injury, as well as increased mortality risk [3–6]. Therefore, heightened vigilance is required in patients who are prone to hypotension. Predicting and preventing intraoperative hypotension is crucial and constitutes a fundamental responsibility of anesthesiologists.

Predicting hypotension during anesthesia induction would significantly enhance the safety of general anesthesia administration. Thus, hypotension prediction remains a major focus of research in anesthesiology, with numerous studies conducted in this area. Various parameters have been used to predict post-induction hypotension, including perfusion index, internal carotid artery blood flow velocity, internal jugular vein diameter, arterial elastance, and cardiac output [7–11]. However, measuring these parameters requires considerable additional effort and resources.

We hypothesized that, in older patients, who often exhibit heightened sympathetic activity, anesthetic-induced sympathetic blockade may increase the risk of severe hypotension. Therefore, preoperative assessment of sympathetic activity may help predict post-induction hypotension. We focused our investigation on nociception monitoring systems, which assess the balance between the sympathetic and parasympathetic nervous systems and generate pain-related indices during general anesthesia using a minimally invasive approach [12, 13]. Heart rate variability, which reflects autonomic nervous system balance, can effectively predict hypotension during anesthetic induction [14, 15]. Based on previous studies demonstrating the association between heart rate variability and post-induction hemodynamic changes, we predicted that pre-induction High Frequency Variability Index (HFVI)/Analgesia Nociception Index (ANI) and Nociception Level (NOL) values would differ between patients who develop hypotension and those who do not, and that these indices could serve as moderate predictors of hypotension during the induction of general anesthesia. We focused specifically on patients aged ≥ 65 years because this population exhibits age-related reductions in parasympathetic tone and baroreflex sensitivity, increasing susceptibility to anesthesia-induced hypotension [1, 2]. Moreover, predicting hypotension in this vulnerable group is of particular clinical importance.

## Methods

### Study design and ethics statement

This study was approved by the Ethics Committee of Yamagata University Hospital on January 18, 2024 (approval number: 2023 − 280). Before commencement, the study was registered as a prospective observational study in the UMIN Clinical Trials Registry (UMIN000053529). Written informed consent was obtained from all participants after a detailed explanation of the study procedures. Patients were informed of their right to withdraw consent at any time without prejudice to their medical care.

### Study participants

The study population was composed of patients aged ≥ 65 years who were scheduled to undergo general anesthesia at Yamagata University Hospital between February and August 2024. This age criterion was established in consideration of the increased risk of hypotension during anesthetic induction in older patients and age-related variations in autonomic nervous system balance [16]. Exclusion criteria included a history of allergy to propofol, remifentanil, and rocuronium; current use of medications affecting autonomic function or heart rate (psychiatric medications or β-blockers); American Society of Anesthesiologists physical status classification (ASA-PS) ≥ 3; technical failure in HFVI/ANI or NOL value calculation; inability to measure blood pressure; and presence of cardiac arrhythmias. Patients receiving β-blockers were excluded because the high-frequency component of heart rate variability may be directly affected, potentially complicating interpretation of the HFVI/ANI and NOL values.

Based on our past institutional data (48 cases) indicating a 47.9% incidence of hypotension with similar induction protocols, the sample size calculation assumed an area under the curve (AUC) of 0.7 for the predictive performance of nociception monitors, with α = 0.05 and 1 - β = 0.8, requiring a minimum of 48 patients. Accounting for a 15% dropout rate, we planned to enroll a minimum of 56 patients. Sample size calculations were performed using R (version 4.3.1; The R Foundation for Statistical Computing, Vienna, Austria).

### Nociception monitoring

Two specific monitoring systems were employed: the HFVI monitor (Mdoloris Medical Systems, Loos, France), which utilizes chest-mounted sensors, and the PMD-200™ (Medasense Biometrics Ltd., Ramat Gan, Israel), which employs digital sensors. Notably, the HFVI is referred to as the ANI in most countries outside Japan. The difference between HFVI and ANI is solely nomenclatural; both use an identical algorithm, including the same band-pass filtering (0.15–0.5 Hz) via wavelet transform, a 64-s moving window with four 16-s sub-windows, and respiratory-based area-under-the-curve analysis of normalized R-R intervals [12]. HFVI was renamed from ANI in 2022 and is displayed on the Root^®^ monitor platform (Masimo Corp.); however, the underlying signal processing remains unchanged. Therefore, throughout this paper, the two are collectively referred to as HFVI/ANI.

The HFVI/ANI is expressed on a scale from 0 to 100, with values closer to 0 indicating greater pain perception, whereas values closer to 100 indicate greater comfort. Manufacturers recommend a target value of 50–70, and many studies have adjusted analgesic administration to maintain values within this range [17–19]. HFVI/ANI comprises two indices: HFVI/ANIi, which represents the average over the past 120 s, and HFVI/ANIm, which represents the average over the past 240 s. The data were updated every 2 s and could subsequently be retrieved later from the recording medium.

The PMD-200™ also displays a value between 0 and 100 for NOL. Unlike the HFVI/ANI, values closer to 0 indicate lower pain perception, whereas values closer to 100 indicate higher pain perception. According to the manufacturer, NOL ≥ 25 is the threshold for pain, and studies have used a protocol to administer analgesics when this value is exceeded [20–22]. In addition, data are updated every 5 s and can be retrieved later from the recording device.

### Anesthesia

No premedication was administered. Peripheral intravenous access was established before admission to the operating room, and lactated Ringer’s solution was administered at approximately 2–3 mL/kg/h. Preoperative fasting status was evaluated in all patients; the last solid meal was dinner on the evening before surgery, and the time of the last clear fluid intake was confirmed individually for each patient. No fluid bolus or preloading was administered before or during induction, and fasting-related deficits were not actively corrected during the observation period. This standardized minimal-fluid approach was intentional and was designed to avoid the confounding effects of fluid loading on post-induction blood pressure and to permit clearer evaluation of the relationship between pre-induction autonomic indices and post-induction hypotension. Upon arrival in the operating room, patients were monitored using standard general anesthesia monitors, supplemented with a Bispectral Index (BIS) sensor placed on the forehead, HFVI/ANI sensors on the anterior chest, and PMD-200 sensors on the left index or middle finger. Pre-induction nociception monitor values, HFVI/ANIi, HFVI/ANIm, and NOL were recorded after 4 min of rest in the supine position, following which, HFVI/ANIi, HFVI/ANIm, and NOL values were recorded immediately before the start of pre-oxygenation. HFVI/ANIi represents the instantaneous value (average over the preceding 120 s), and HFVI/ANIm represents the mean value (average over the preceding 240 s), both captured at this single time point. NOL values were recorded simultaneously. Patients were instructed to maintain regular breathing patterns to avoid bradypnea (< 9 breaths/min). Following pre-oxygenation, remifentanil was administered at 0.2 µg/kg/min based on ideal body weight (IBW) for 2 min, followed by a rapid intravenous bolus administration of propofol at 2 mg/kg based on actual body weight. Loss of consciousness was confirmed by absence of response to verbal command (calling the patient’s name) and loss of the eyelash reflex, after which rocuronium at 0.6 mg/kg (IBW) was administered and mask ventilation was performed using a tidal volume of approximately 8 mL/kg (IBW). Noninvasive blood pressure was measured at 1-min intervals for 5 min following propofol administration, with mean arterial pressure (MAP) recorded throughout this period.

Patient characteristics, including sex, height, weight, ASA-PS, estimated glomerular filtration rate, medications, comorbidities, baseline heart rate, and MAP (measured in the ward before operating room admission), and fasting duration, were obtained from electronic medical records.

Hypotension was defined as MAP < 65 mmHg [14]. Patients who developed a MAP < 65 mmHg within 5 min of propofol administration were classified into the hypotension group. If hypotension was observed, vasopressors were administered, and monitoring was discontinued before completion of the 5-min observation period. For patients whose MAP level fell below 65 mmHg during the 5-min observation period and who were administered vasopressors, the last MAP value recorded immediately before the administration of vasopressor was carried forward to subsequent time points. Patients maintaining MAP ≥ 65 mmHg throughout the 5 min were classified into the nonhypotension group.

### Study outcomes

The primary outcome was the predictive performance of nociception monitor values (HFVI/ANIi, HFVI/ANIm, NOL) for hypotension during general anesthesia induction, evaluated using receiver operating characteristic (ROC) curve analysis and corresponding AUC values. Secondary outcomes included between-group comparisons of age, weight, comorbidities, baseline heart rate and MAP, pre-induction nociception monitor values, propofol dose, and fasting duration. Multivariable regression analysis was performed to identify independent predictors of hypotension. Additionally, to quantify the cumulative burden, including the severity and duration of post-induction hypotension, the area under the MAP < 65 mmHg (AUT-MAP < 65 mmHg; hereafter referred to as AUT-65) was calculated for each patient. This is the sum of the differences between 65 mmHg and the measured MAP at each 1-min time point (unit: mmHg·min), with a value of 0 when the MAP was ≥ 65 mmHg [23, 24]. The association between pre-induction nociception index values and AUT-65 was evaluated using Spearman rank correlation analysis, because AUT-65 values were not normally distributed. Additionally, to examine the temporal pattern of MAP changes following anesthesia induction, patients were stratified into two groups based on the optimal cutoff values derived from ROC curve analysis: a group greater than the cutoff value and a group below the cutoff value for each nociceptive monitor. MAP values at baseline (measured in the ward) and at 1, 2, 3, 4, and 5 min after propofol administration were compared between groups using a mixed-effects model for repeated measures (REML), with group as the between-subject factor and time as the within-subject factor. The Geisser–Greenhouse correction was applied to account for potential violations of sphericity. When significant main effects or interactions were identified, Šidák’s multiple comparisons test was used to compare groups at each individual time point.

### Statistical analysis

Continuous variables were tested for normality using the Shapiro–Wilk test. Normally distributed data are summarized as mean ± standard deviation and were compared using unpaired *t*-tests. Non-normally distributed data are presented as median [interquartile range] and were compared using Mann–Whitney U tests. Categorical variables were analyzed using chi-square or Fisher’s exact tests. Multivariable logistic regression analysis was conducted to identify independent predictors of hypotension. The predictive performance of nociception monitoring was evaluated using ROC curve analysis, with optimal cutoff values determined using the Youden index. Spearman rank correlation coefficients were used to assess the associations between pre-induction nociception index values (HFVI/ANIm, HFVI/ANIi, NOL) and AUT-65. Mixed-effects models for repeated measures were used to analyze the time course of MAP changes, with Šidák’s correction for multiple comparisons at individual time points. Data analysis was performed using GraphPad Prism (version 10.3.1; GraphPad Software, San Diego, CA, USA), with statistical significance set at *p* < 0.05.

## Results

**Fig. 1:**
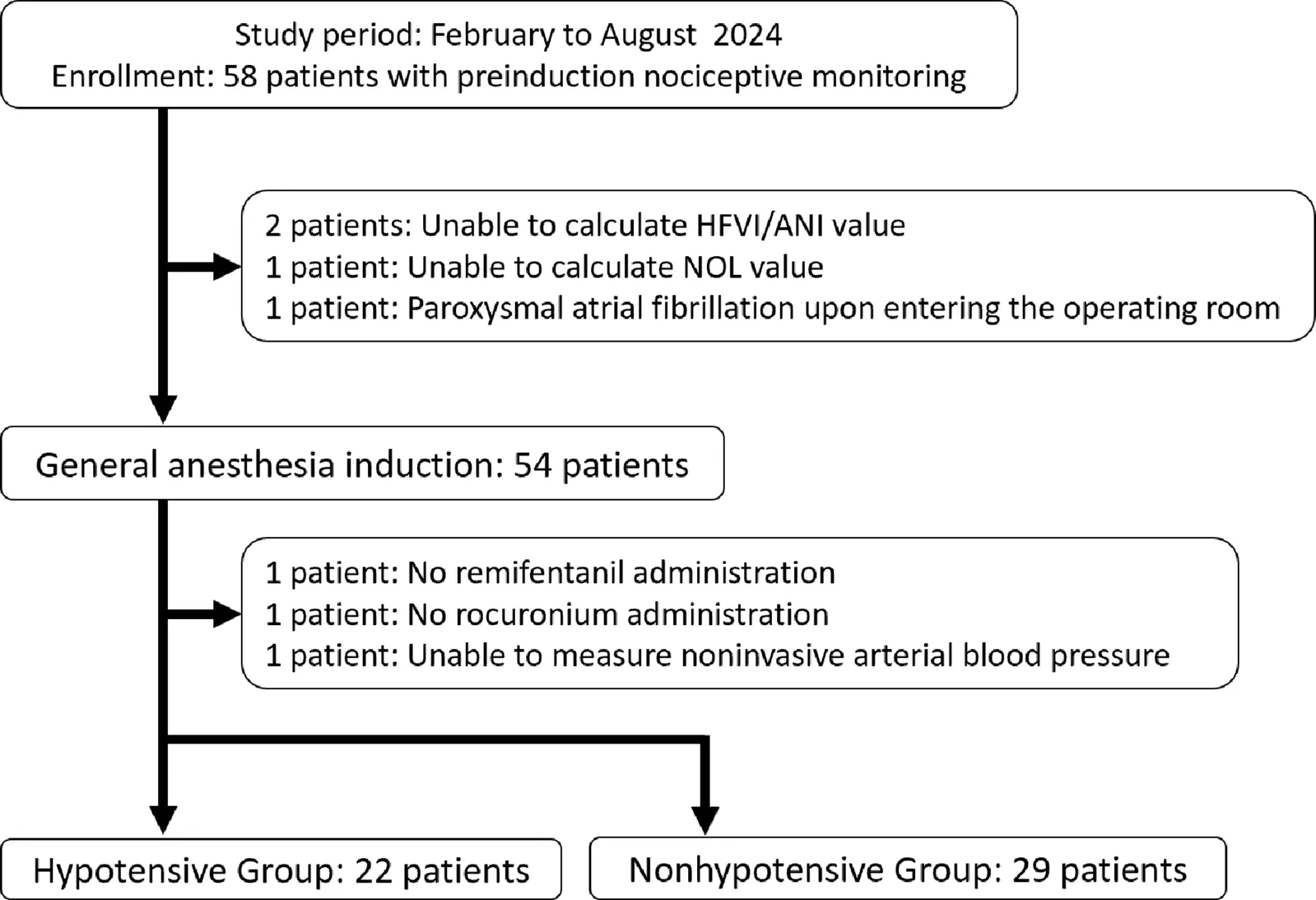
Patient Flow Diagram. Flow of patient enrollment, exclusions, and classification into hypotension and nonhypotension groups

Of the 58 enrolled patients, 7 were excluded from the final analysis: 2 because of failure of HFVI/ANI value calculation and 1 because of NOL value calculation (in all three cases, the monitors did not output values, most likely because of poor signal quality rather than arrhythmias); 1 because of paroxysmal atrial fibrillation on entering the operating room; 1 because blood pressure could not be measured after anesthetic induction; and 2 because of protocol violations (omission of remifentanil in one case and rocuronium in another). The remaining 51 patients were included in the final analysis, with 22 classified into the hypotension group and 29 into the nonhypotension group (Fig. 1).

Baseline demographic and clinical characteristics did not differ significantly between the groups (Table 1). Compared with the nonhypotension group, the hypotension group demonstrated significantly lower HFVI/ANIi values (58.7 ± 11.1 vs. 69.0 ± 15.1, *p* = 0.010) and higher NOL values (17.2 ± 9.1 vs. 10.7 ± 8.8, *p* = 0.013). However, HFVI/ANIm values did not differ significantly between the groups (59.2 ± 9.4 vs. 66.4 ± 13.4, *p* = 0.054) (Table 2). The doses of propofol and remifentanil used for anesthetic induction followed the protocol in all cases.

**Table 1:** Comparison of Patient Characteristics

	Hypotension Group (*n* = 22)	Nonhypotension Group (*n* = 29)	*p*-value
Age (years)	73.4 ± 4.1	72.0 ± 4.7	0.269
Sex (male/female)	6/16	13/16	0.250
Height (cm)	155.9 ± 8.6	159.0 ± 9.6	0.244
Weight (kg)	59.4 ± 9.9	60.4 ± 11.6	0.746
*Comorbidities*			
Cardiovascular disease, n (%)	0 (0)	1 (3.5)	> 0.999
Cerebrovascular disease, n (%)	0 (0)	0 (0)	−
Diabetes mellitus, n (%)	4 (18.2)	6 (20.7)	> 0.999
COPD, n (%)	3 (13.6)	1 (3.5)	0.303
Hypertension, n (%)	15 (68.2)	17 (58.6)	0.484
eGFR (mL/min)	75.5 ± 13.0	74.3 ± 20.0	0.806
*Antihypertensive Medication*			
Ca channel blocker, n (%)	8 (36.4)	13 (44.8)	0.543
ARB, n (%)	6 (27.3)	7 (24.1)	0.799
Diuretics, n (%)	3 (13.6)	0 (0)	0.074
*ASA-PS*			
I, n (%)	0 (0)	4 (13.8)	0.124
II, n (%)	22 (100)	25 (86.2)	
*Surgical Timing*			
Morning, n (%)	19 (86.4)	19 (65.5)	0.115
Afternoon, n (%)	3 (13.6)	10 (34.5)	
Fasting duration (min)	150 [150–168.8]	150 [150.0–250.0]	0.082

Values are presented as mean ± standard deviation or median [interquartile range]

COPD, chronic obstructive pulmonary disease; ARB, angiotensin II receptor blocker; ASA-PS, American Society of Anesthesiologists Physical Status; eGFR, estimated glomerular filtration rate

**Table 2:** Comparison of Surgical Information and Nociceptive Monitor Values Between Groups

	Hypotension Group (n = 22)	Nonhypotension Group (n = 29)	p-value
*Pre-induction Values*			
Heart rate (bpm)	70 ± 9.0	72 ± 12.0	0.681
MAP (mmHg)	95.5 ± 6.8	96.4 ± 10.8	0.738
HFVI/ANIm	59.2 ± 9.4	66.4 ± 13.4	0.054
HFVI/ANIi	58.7 ± 11.1	69.0 ± 15.1	0.010
NOL	17.2 ± 9.1	10.7 ± 8.8	0.013
*Post-induction*			
Heart rate (bpm)	63.4 ± 7.8	61.6 ± 10.8	0.488
MAP (mmHg)	60.6 ± 3.2	73.5 ± 5.4	< 0.0001
Minimum BIS Values	37.9 ± 7.8	37.5 ± 7.7	0.859

Values are presented as mean ± standard deviation or median [interquartile range]

MAP, mean arterial pressure; BIS, Bispectral Index; HFVI/ANIm and HFVI/ANIi, mean and instantaneous High Frequency Variability Index/Analgesia Nociception Index; NOL, Nociception Level

In the multivariable logistic regression analysis, we incorporated age and baseline MAP (previously established predictors in the literature [2, 15, 25]), together with preoperative HFVI/ANIi and NOL values, which had shown significant differences in univariable analysis. None of these variables emerged as independent predictors of hypotension during general anesthesia induction (HFVI/ANIi: odds ratio 0.96, 95% confidence interval [CI] 0.91–1.01, *p* = 0.110; NOL: odds ratio 1.06, 95% CI 0.98–1.16, *p* = 0.136) (Table 3).

**Table 3:** Multivariate Logistic Regression Analysis of Independent Variables for Hypotension During General Anesthesia Induction

Variables	Adjusted Odds Ratio (95% CI)	*p*-value
Age	1.10 (0.95–1.28)	0.208
Baseline MAP	0.97 (0.90–1.04)	0.452
HFVI/ANIi	0.96 (0.91–1.01)	0.110
NOL	1.06 (0.98–1.16)	0.136

MAP, mean arterial pressure; HFVI/ANIi, instantaneous High Frequency Variability Index/Analgesia Nociception Index; NOL, Nociception Level; CI, confidence interval

**Fig. 2:**
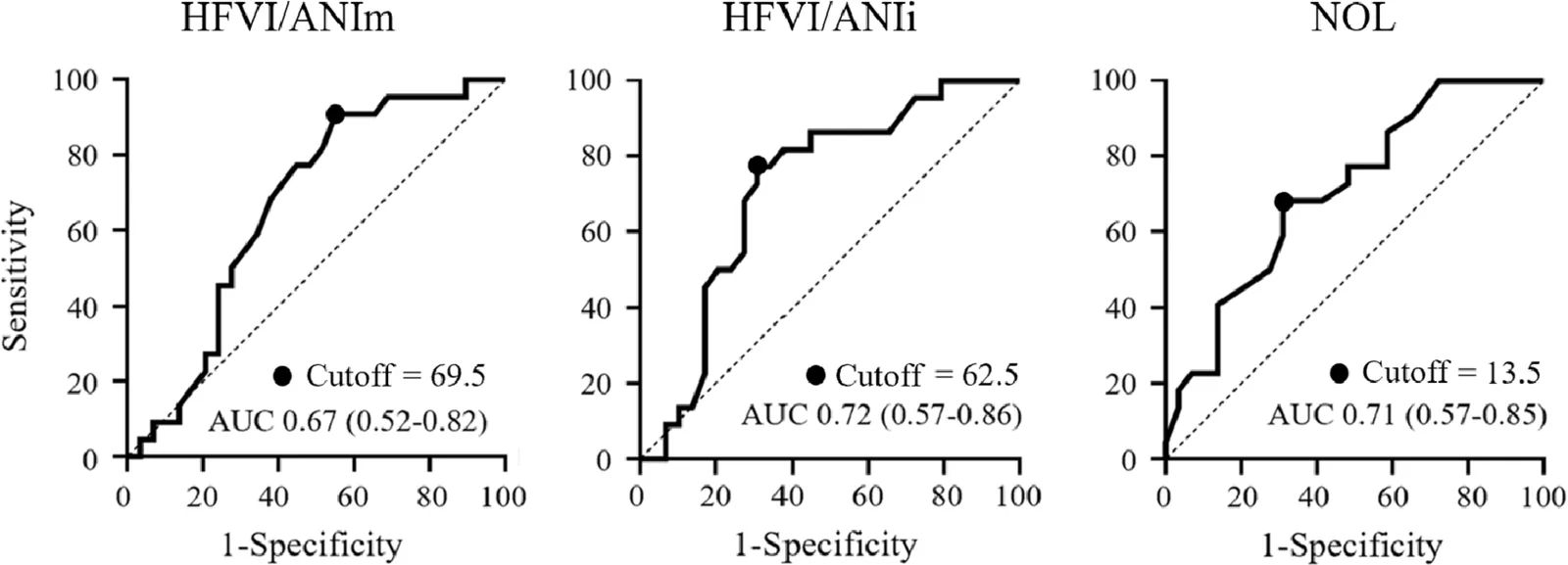
Receiver Operating Characteristic (ROC) Curves for Predicting Post-induction Hypotension Using Nociception Monitors. The curves show the predictive performance of nociception indices measured before anesthesia induction: the mean and High Frequency Variability Index/Analgesia Nociception Index (HFVI/ANIm), the instantaneous HFVI/ANI (HFVI/ANIi), and the Nociception Level (NOL). The optimal cutoff values were 69.5 for ANIm, 62.5 for ANIi, and 13.5 for NOL. The area under the curve (AUC) with corresponding 95% confidence intervals is indicated for each monitor

ROC curve analysis yielded the following results for each nociception monitor parameter: HFVI/ANIm showed an AUC of 0.67 with an optimal cutoff value of 69.5 (sensitivity 90.9%, specificity 44.8%); HFVI/ANIi demonstrated an AUC of 0.72 with a cutoff value of 62.5 (sensitivity 77.3%, specificity 69.0%); and NOL exhibited an AUC of 0.71 with a cutoff value of 13.5 (sensitivity 68.2%, specificity 68.9%) (Table 4; Fig. 2).

**Table 4:** Predictive Performance of Nociceptive Monitoring for Post-induction Hypotension

Predictor	Cutoff value	AUC (95% CI)	*p*-value	Sensitivity	Specificity
HFVI/ANIm	69.5	0.67 (0.52–0.82)	0.0440	90.9%	44.8%
HFVI/ANIi	62.5	0.72 (0.57–0.86)	0.0092	77.3%	69.0%
NOL	13.5	0.71 (0.57–0.85)	0.0110	68.2%	68.9%

HFVI/ANIm and HFVI/ANIi, mean and instantaneous High Frequency Variability Index/Analgesia Nociception Index; NOL, Nociception Level; AUC, area under the curve; CI, confidence interval

**Fig. 3:**
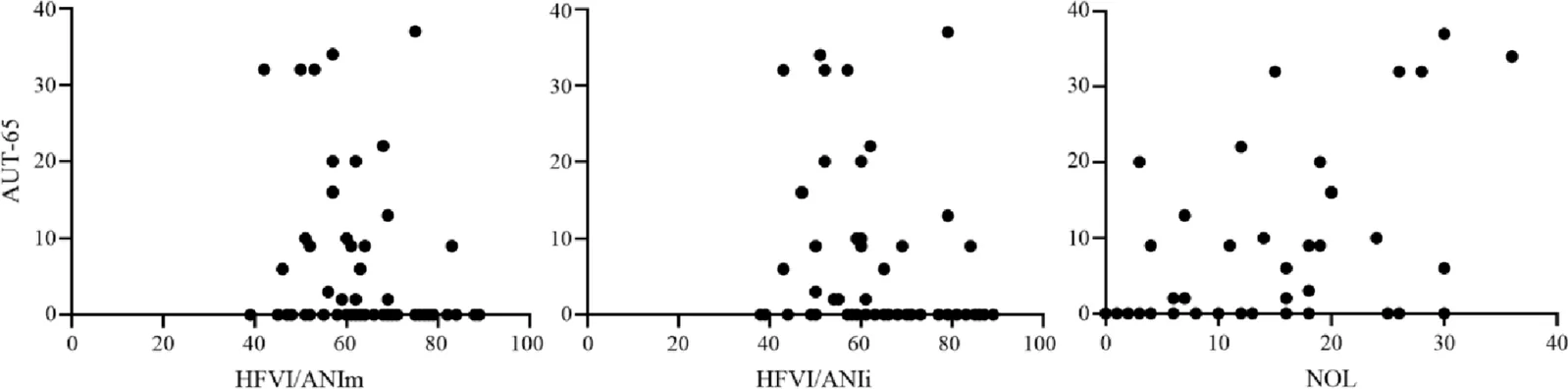
Correlation Between Pre-induction Nociception Index Values and the Area Under the Threshold of Mean Arterial Pressure Below 65 mmHg (AUT-65). Scatter plots showing the relationship between AUT-65 (mmHg·min) and pre-induction nociception index values: (**A**) HFVI/ANIm (Spearman rho = − 0.29, *p* = 0.038), (**B**) HFVI/ANIi (rho = − 0.35, *p* = 0.013), and (**C**) NOL (rho = 0.47, *p* = 0.0005). AUT-65 quantifies the cumulative burden of hypotension by integrating the depth and duration of mean arterial pressure below 65 mmHg over the 5-min post-induction observation period. Higher AUT-65 values indicate more severe and sustained hypotension

The AUT-65 analysis revealed that the hypotension group had a median AUT-65 value of 10.0 mmHg·min (range: 2–37); as expected, all patients in the nonhypotension group exhibited an AUT-65 value of 0 mmHg·min. Pre-induction nociception index values were significantly correlated with AUT-65: HFVI/ANIm demonstrated a weak negative correlation (Spearman rho = − 0.29, 95% CI − 0.53 to − 0.01, *p* = 0.038), HFVI/ANIi demonstrated a moderate negative correlation (rho = − 0.35, 95% CI − 0.57 to − 0.07, *p* = 0.013), and NOL exhibited a moderate positive correlation (rho = 0.47, 95% CI 0.22 to 0.66, *p* = 0.0005) (Table 5; Fig. 3). These findings indicate that patients with lower pre-induction HFVI/ANIi values and higher NOL values experienced not only a higher incidence of hypotension but also a greater cumulative hypotensive burden following anesthesia induction.

**Table 5:** Spearman Rank Correlation Between Pre-induction Nociception Index Values and AUT-65

Predictor	Spearman rho	95% CI	*p*-value
HFVI/ANIm	− 0.29	− 0.53 to − 0.01	0.038
HFVI/ANIi	− 0.35	− 0.57 to − 0.07	0.013
NOL	0.47	0.22 to 0.66	0.0005

AUT-65, area under mean arterial pressure below 65 mmHg; HFVI/ANIm and HFVI/ANIi, mean and instantaneous High Frequency Variability Index/Analgesia Nociception Index; NOL, Nociception Level; CI, confidence interval

**Fig. 4:**
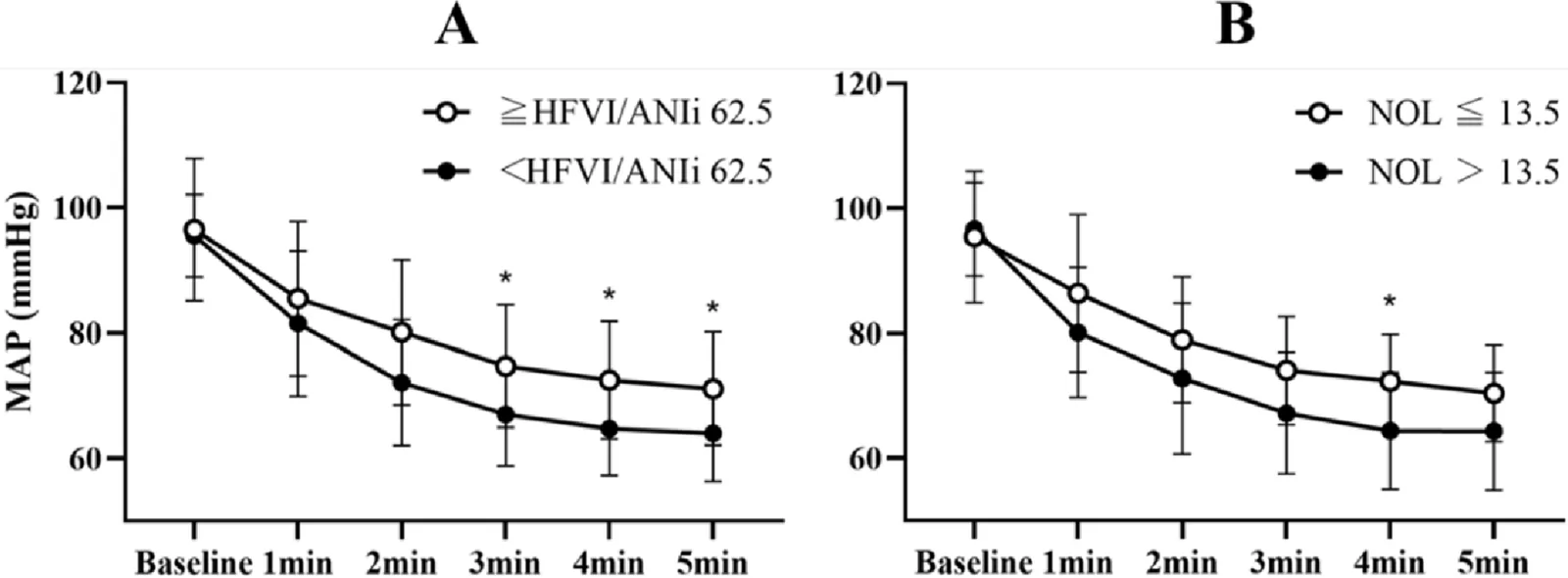
Time Course of Mean Arterial Pressure Changes Following Anesthesia Induction Stratified by Pre-induction Nociception Index Values. Mean arterial pressure (MAP) values at baseline and at 1, 2, 3, 4, and 5 min after propofol administration are shown as mean ± standard deviation. (**A**) Patients stratified by pre-induction HFVI/ANIi values: HFVI/ANIi < 62.5 (●, n = 26) versus HFVI/ANIi ≥ 62.5 (○, n = 25). The mixed-effects model showed significant effects for time (p < 0.0001) and group (p = 0.010), with the interaction approaching significance (p = 0.065). (**B**) Patients stratified by pre-induction NOL values: NOL > 13.5 (●, n = 24) versus NOL ≤ 13.5 (○, n = 27). The mixed-effects model showed significant effects for time (p < 0.0001), group (p = 0.021), and time-by-group interaction (p = 0.022). Asterisks indicate significant between-group differences at individual time points by Šidák’s multiple comparisons test (*p < 0.05)

The time-course analysis of the MAP changes following anesthesia induction is shown in Fig. 4. When patients were stratified by pre-induction HFVI/ANIi values using the optimal cutoff of 62.5, the mixed-effects model revealed significant main effects for time (*p* < 0.0001) and group (*p* = 0.010), although the interaction between time and group did not reach statistical significance (*p* = 0.065). Šidák’s multiple comparisons test demonstrated that the HFVI/ANIi < 62.5 group (*n* = 26) had significantly lower MAP values than the HFVI/ANIi ≥ 62.5 group (*n* = 25) at 3 min (67.0 ± 8.2 vs. 74.8 ± 9.7 mmHg, *p* = 0.022), 4 min (64.8 ± 7.5 vs. 72.5 ± 9.4 mmHg, *p* = 0.013), and 5 min (64.0 ± 7.6 vs. 71.2 ± 9.0 mmHg, *p* = 0.022) after propofol administration, whereas no significant differences were observed at baseline, 1 min, or 2 min (Fig. 4A).

Similarly, when patients were stratified by pre-induction NOL values using the cutoff of 13.5, the mixed-effects model demonstrated significant main effects for time (*p* < 0.0001) and group (*p* = 0.021), as well as a significant time-by-group interaction (*p* = 0.022), indicating that the pattern of MAP decline differed between the two groups. The NOL > 13.5 group (*n* = 24) exhibited a more rapid decline in MAP compared with the NOL ≤ 13.5 group (*n* = 27), with a significant between-group difference at 4 min (64.4 ± 9.3 vs. 72.3 ± 7.5 mmHg, *p* = 0.013) (Fig. 4B).

## Discussion

Consistent with our hypothesis, pre-induction HFVI/ANI and NOL values differed significantly between patients who developed post-induction hypotension and those who did not. This study demonstrated a significant association between pre-induction nociception index values and the occurrence of post-induction hypotension during general anesthesia. Specifically, pre-induction HFVI/ANIi values below 62.5 and NOL values above 13.5 were associated with an increased likelihood of developing post-induction hypotension. However, given the observational design and multivariable analysis results, these findings should be interpreted as associations rather than causal or independently predictive relationships.

Importantly, the AUT-65 analysis extended our findings beyond a binary hypotension classification by demonstrating that pre-induction nociception indices were significantly correlated with the cumulative burden of post-induction hypotension. The use of AUT-65, a metric that integrates the depth and duration of hypotension below a predefined threshold, has been established in perioperative hemodynamic research [6, 23, 24]. In the present study, we adopted a threshold of 65 mmHg, consistent with our primary hypotension definition. The significant correlations between pre-induction HFVI/ANIi (rho = − 0.35) and NOL (rho = 0.47) values and AUT-65 suggest that these indices reflect not only the likelihood of developing hypotension but also its severity. Furthermore, the AUT-65 analysis revealed that these indices were associated with the cumulative burden of hypotension, indicating that pre-induction autonomic status may be related to the magnitude of post-induction hemodynamic changes. Although the present study measured nociception indices only at a single pre-induction time point, these values were associated with the subsequent temporal pattern of blood pressure decline, as captured by AUT-65. Future studies employing continuous nociception index monitoring throughout the induction period could further elucidate the temporal dynamics of autonomic changes in relation to hemodynamic instability. The time-course analysis further substantiated these findings by demonstrating that patients stratified by pre-induction nociception index cutoff values exhibited divergent MAP trajectories following anesthesia induction, despite similar baseline blood pressures. When stratified by HFVI/ANIi, the low-value group showed significantly lower MAP from 3 min onward, whereas stratification by NOL revealed a significant time-by-group interaction (*p* = 0.022), indicating that patients with higher pre-induction NOL values experienced a distinctly more rapid hemodynamic decline. These temporal analyses complement the AUT-65 findings and provide a more granular understanding of how pre-induction autonomic status influences the dynamics of post-induction blood pressure changes.

Heart rate variability parameters, particularly reduced high-frequency components, have been associated with post-induction hypotension [26, 27]. However, to our knowledge, no previous study has specifically evaluated the discriminative ability of commercially available nociception monitoring devices (HFVI/ANI and NOL), which provide real-time processed indices of autonomic balance, for predicting hypotension during general anesthesia induction. The present study extends the existing heart rate variability-based literature by demonstrating that clinically accessible, device-derived indices show moderate discriminative performance. Notably, HFVI/ANI has been reported to predict hypotension during spinal anesthesia [28]. In that study of combined spinal-epidural labor analgesia, the mean HFVI/ANI values were 58.6 ± 1.4 in the hypotensive group and 70.6 ± 1.1 in the normotensive group. Remarkably, our study in patients undergoing general anesthesia demonstrated similar HFVI/ANIi values (58.7 ± 11.1 vs. 69.0 ± 15.1), with significantly lower values also observed in the hypotension group (*p* = 0.010). This consistency suggests that HFVI/ANI values around 59 may represent a critical threshold for predicting hypotension risk, regardless of whether neuraxial or general anesthesia is employed.

Previous research employing heart rate variability for hypotension prediction has shown that patients who developed post-induction hypotension demonstrated significantly lower high-frequency components, indicating parasympathetic involvement [26, 28]. Additional studies reported higher low-frequency to high-frequency ratios in the hypotension group [27, 28], suggesting sympathetic nervous system involvement in post-induction hypotension. Although multivariable analysis did not identify them as independent predictors, both HFVI/ANI—which evaluates parasympathetic activity through high-frequency components of heart rate variability—and NOL—which assesses autonomic balance through multiple parameters, including heart rate variability, skin conductance, and photoplethysmography—demonstrated useful predictive capability as evidenced by their ROC curve analyses. Furthermore, these nociception indices may offer potential clinical utility as screening tools. They are applicable not only to pre-anesthesia induction and intraoperative pain assessment, but also to preoperative outpatient screening. Currently, pre-induction assessment using HFVI/ANI and NOL may enable risk stratification before anesthesia induction. Patients identified as high-risk based on HFVI/ANIi values < 62.5 or NOL values > 13.5 could be considered for preemptive interventions, such as prophylactic low-dose vasopressor administration (e.g., phenylephrine bolus or norepinephrine infusion at induction), fluid preloading, or reduced doses of induction agents. The integration of these monitors into the pre-induction workflow could support a more individualized approach to anesthesia induction in older patients.

Regarding the clinical feasibility of implementing the proposed cutoff values, we acknowledge that the current protocol requires a 4-min pre-induction recording period for HFVI/ANIm values, which may limit real-time usability. However, HFVI/ANIi, which showed superior discriminative performance (AUC = 0.72), requires only a 120-s averaging window. In practice, HFVI/ANI and NOL monitors can be applied during standard pre-induction monitoring, allowing continuous observation of values on the bedside display. A practical workflow could involve attaching the monitors upon arrival in the operating room, allowing values to stabilize during routine preparation (positioning, standard monitoring), and reviewing the HFVI/ANIi and NOL values immediately before induction—without requiring any additional dedicated recording time. Future studies should explore whether shorter observation windows or real-time rolling averages can maintain discriminative performance while further improving workflow integration.

Our findings revealed significant differences in HFVI/ANIi but not HFVI/ANIm between the hypotension and nonhypotension groups. This disparity may be attributed to their different measurement windows: HFVI/ANIi represents the mean of the preceding 120 s, whereas HFVI/ANIm represents the mean of the preceding 240 s. Although measurements were obtained 4 min after patients assumed the supine position to accommodate HFVI/ANIm characteristics, this duration may have been insufficient for complete autonomic equilibration following the positional change. The higher AUC for HFVI/ANIi (0.72) than for HFVI/ANIm (0.67) suggests that HFVI/ANIi’s shorter measurement window might more accurately reflect immediate autonomic status. NOL, which does not rely on time-averaged measurements, demonstrated predictive capability similar to that of HFVI/ANIi, with both parameters potentially providing a more accurate assessment of autonomic status than HFVI/ANIm.

The absence of significant between-group differences in patient characteristics and anesthetic factors, combined with significant differences in HFVI/ANIi and NOL values, enabled clearer evaluation of the relationship between nociception indices and the occurrence of hypotension. However, multivariable analysis incorporating previously established risk factors (age and baseline MAP) did not identify independent predictors of hypotension. This discrepancy from previously reported findings [2, 25] might be attributed to our age-restricted cohort (≥ 65 years) compared to studies with broader age distributions. Additionally, our sample size calculation was based on the predictive performance of nociception indices rather than on requirements for multivariable analysis, which may have resulted in insufficient statistical power to identify independent predictors.

Previous research evaluating HFVI/ANI in healthy young volunteers (mean age: 17 years) reported mean awakening HFVI/ANI values of 90.1 [29], considerably higher than the mean HFVI/ANIm of 63.6 observed in our cohort. This disparity likely reflects age-related differences in autonomic balance [16] and the impact of the potentially stressful operating room environment. The lower HFVI/ANI values observed in our study reasonably suggest reduced parasympathetic activity and heightened tension, consistent with the clinical context.

Our study has some limitations. First, although circadian variations in heart rate variability are well-documented [30], our study did not explicitly account for this factor. Although no significant differences in measurement timing were observed between groups, detailed time-stratified analyses remain a subject for future investigation. Second, our age-restricted inclusion criterion (≥ 65 years) limits the generalizability of our findings to younger populations. However, this focus on older patients is clinically relevant given their increased susceptibility to anesthesia-induced hypotension and associated complications [31]. Third, our sample size may have been insufficient to identify independent predictors through multivariable analysis. Fourth, although nociceptive monitoring itself is widespread, this was a single-center study, which may limit external generalizability. Furthermore, institution-specific clinical practices may have influenced hemodynamic responses during the induction period, and these factors may limit the generalizability of our findings to other institutions with different clinical workflows. Nevertheless, the significant differences observed in HFVI/ANIi and NOL values between groups, together with their respective AUCs of 0.72 and 0.71, represent novel findings that suggest their potential utility in hypotension prediction. Fifth, as a non-blinded observational study, the attending anesthesiologists were aware of the nociception monitor values, which could have influenced clinical decision-making and introduced performance bias. However, the anesthesia induction protocol was standardized with fixed doses of propofol and remifentanil, limiting the scope for such bias during the observation period. Sixth, the AUT-65 analysis was limited by the use of noninvasive blood pressure measurements at 1-min intervals rather than continuous arterial pressure monitoring. Continuous monitoring would have provided a more precise estimate of the hypotensive burden. Additionally, because vasopressors were administered upon detection of hypotension, the approach used for subsequent time points provides a conservative estimate of the true AUT-65, as blood pressure would likely have continued to decline without intervention. Seventh, although exclusion of patients receiving β-blockers was necessary to avoid pharmacological confounding of HRV-derived indices, this criterion limits the generalizability of our findings, as patients receiving β-blockers represent a clinically important subgroup within the older surgical population. The predictive utility of HFVI/ANI and NOL in patients receiving β-blockers warrants further dedicated investigation.

In conclusion, this study demonstrates a significant association between pre-induction nociception indices (HFVI/ANI and NOL) and post-induction hypotension during general anesthesia in patients aged ≥ 65 years. Pre-induction HFVI/ANIi values below 62.5 and NOL values above 13.5 were associated with an increased incidence of post-induction hypotension. Furthermore, AUT-65 analysis revealed that these indices were associated with the cumulative burden of hypotension, indicating that pre-induction autonomic status may be related to the magnitude of post-induction hemodynamic changes. Although these parameters were not identified as independent predictors in multivariable analysis, and a causal relationship cannot be established from this observational study, nociception monitors may serve as adjunctive screening tools to identify patients at higher risk of anesthesia-induced hypotension. Prospective interventional studies are needed to determine whether risk stratification based on pre-induction nociception index values can guide preventive strategies and ultimately reduce the incidence and severity of post-induction hypotension.

## Data Availability

The datasets generated and analyzed during the current study are not publicly available due to patient privacy considerations but are available from the corresponding author upon reasonable request and with appropriate institutional ethical approval.
